# Platelet Activation on Lung Function During Ex Vivo Lung Perfusion, Lung Transplantation, and the Role of Antiplatelet Therapy: A Narrative Review

**DOI:** 10.1097/TXD.0000000000001855

**Published:** 2025-08-22

**Authors:** Ryaan EL-Andari, Jimmy J.H. Kang, Nicholas M. Fialka, Jason Weatherald, Parnian Alavi, Nadia Jahroudi, Darren H. Freed, Jayan Nagendran

**Affiliations:** 1 Division of Cardiac Surgery, Department of Surgery, University of Alberta, Edmonton, AB, Canada.; 2 Division of Pulmonary Medicine, Faculty of Medicine and Dentistry, University of Alberta, Edmonton, AB, Canada.; 3 Faculty of Medicine and Dentistry, University of Alberta, Edmonton, AB, Canada.

## Abstract

**Background.:**

Ischemia/reperfusion injury after lung transplantation is a significant cause of morbidity. In the realm of ex vivo lung perfusion (EVLP), inflammation, edema formation, and reduced compliance have limited the durability of EVLP. Previous evidence has suggested that platelet activation and thrombosis may play a role in both conditions.

**Methods.:**

A literature search of PubMed and Embase was conducted, including all articles describing all human or animal investigations of platelet activation or the use of antiplatelet agents in the settings of EVLP or lung transplantation. Articles published from database inception to July 15, 2024, were analyzed.

**Results.:**

In total, 9 studies were included in the review. Studies on EVLP have found an association between platelet activation and adverse effects on lung function, whereas in lung transplantation, platelet activation appears to play a role in primary graft dysfunction. In both settings, the inhibition of platelets ameliorated these effects.

**Conclusions.:**

Platelet activation in EVLP and lung transplantation results in distal arterial thrombosis and has been associated with graft dysfunction. The use of antiplatelet agents in the included studies was associated with reduced lung injury and improved lung function on EVLP or during lung transplantation.

## Graphical abstract

**Figure:**
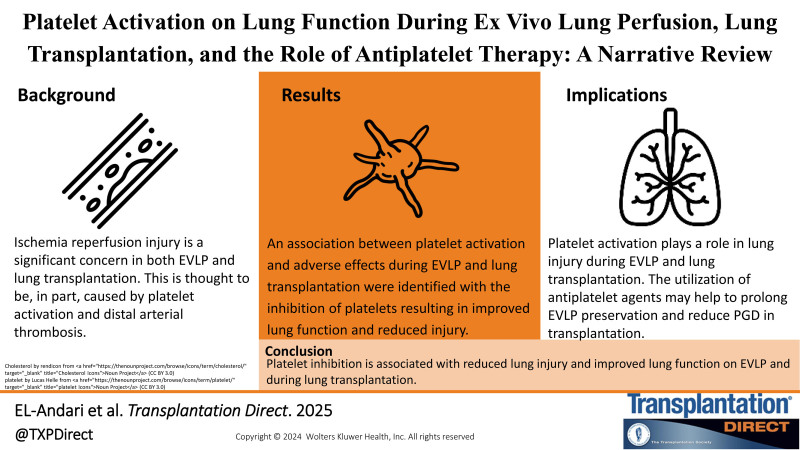

## INTRODUCTION

Lung transplantation remains the gold standard therapy for patients with end-stage lung disease. Outcomes after lung transplantation have continually improved during recent decades, largely owing to improvements in surgical technique and postoperative medical therapy^.1,2^ One of the largest limitations remaining in lung transplantation is the availability of donor organs, with considerable waitlist mortality across programs.^3-7^ Furthermore, ischemia/reperfusion injuries may occur after transplantation, leading to tissue injury, primary graft dysfunction (PGD), accelerated rejection, and increased rates of mortality.^8-12^ Platelet activation, due to inflammation and endothelial injury, has been proposed to be involved in this mechanism during transplantation.^8,9,11^

Ex vivo lung perfusion (EVLP) has been developed and refined to create an invaluable tool in the setting of lung transplantation. EVLP has been able to prolong the duration of organ preservation, allow for evaluation of donor lungs before transplantation, and has even been able to facilitate recovery of function in marginal donor lungs such that they are suitable for transplantation.^13-18^ EVLP has the potential to address the issue of availability in lung transplantation by expanding the donor pool with recovery of function and prolonged preservation times. Three current EVLP protocols are most commonly used. The Toronto Protocol involves an acellular perfusate with Steen solution and a centrifugal pump. Cardiac output is set to 40% and a closed left atrium is used.^4,18-22^ The Organ Care System (OCS; OCS Lung, Transmedics, Andover, MA) is a portable device that uses a cellular perfusate with OCS solution and red blood cells, targeting a hematocrit of 15%–25%, open left atrium, and cardiac output of 2–2.5 L/min.^4,16,18,21,22^ Finally, the Lund protocol perfuses lungs at 100% of cardiac output with an open left atrium and cellular perfusate, using Steen solution and red blood cells, targeting a hematocrit of 14%.^18,22^

EVLP today continues to be limited in several respects. One such limitation is the durability of EVLP. EVLP has largely been limited to <24 h, with select reports extending past this duration.^23-26^ Although EVLP has shown utility in recovering donor organs in the short term by improving the function of marginal donor lungs,^13,27^ prolonged durations of EVLP result in adverse changes, such as edema formation, elevating lactate, and increasing ventilatory resistance signal worsening lung function and a resultant upper limit to the duration of EVLP.^14,27^ Several factors have been proposed to contribute to these findings, including ischemia/reperfusion injuries.

One such factor is platelet activation and thrombosis of the distal vessels of the donor organ during organ procurement and after implantation.^10^ Although platelet activation has been identified in the setting of EVLP, no consensus has been reached regarding the use of antiplatelet agents during EVLP. Similarly, there have been limited investigations into the use of antiplatelet agents in lung transplantation. Herein, we perform a narrative review of antiplatelet use in lung transplantation and EVLP to consolidate the available information on this topic. We seek to identify evidence of benefit from antiplatelet use and any paucity of data in this field of study.

## MATERIALS AND METHODS

### Data Sources

A literature search of PubMed and Embase was conducted by 2 authors. The search terms used individually or in combination for this review were “lung transplantation, ex situ lung perfusion, ex vivo lung perfusion, ESLP, EVLP, antiplatelets, platelets, aspirin, ASA, ticagrelor, clopidogrel, prasugrel, cangrelor, Plavix, Brilinta” individually or in combination. This review included all English language articles describing all human or animal investigations of platelet activation or the use of antiplatelet agents in the settings of EVLP or lung transplantation. Notably, both the terms ex situ lung perfusion and EVLP have been used in the literature on this topic. Although we used EVLP in this study to maintain consistency, both ex situ lung perfusion and EVLP were used in the search to identify articles to avoid missing relevant articles.

### Study Selection

Randomized controlled trials, prospective, retrospective, and observational studies, as well as animal studies describing the impact of platelet activation, thrombosis, or the use of antiplatelet agents in the setting of lung transplantation or EVLP were included. The exclusion criteria for this review included case reports, editorials, and abstracts without an associated full text.

### Outcomes

Outcomes of interest included measures of platelet aggregation, thrombus formation, obliterative bronchiolitis, and lung function in either EVLP or transplantation settings, including oxygenation, lung compliance, pulmonary vascular resistance (PVR), and edema formation. Other outcomes related to organ ischemia or ischemia/reperfusion injuries, such as PGD and obliterative bronchiolitis, were also included. Given the expected heterogeneity between studies, including small animal, large animal, and human models, there was no predetermined plan to perform aggregate analyses on the available data.

### Risk of Bias

Risk of bias was assessed for the included animal studies using SYRCLE’s risk of bias tool for animal studies.^28^ The risk of bias assessment includes questions regarding selection bias, performance bias, detection bias, attrition bias, and reporting bias.

## RESULTS

**FIGURE 1.**
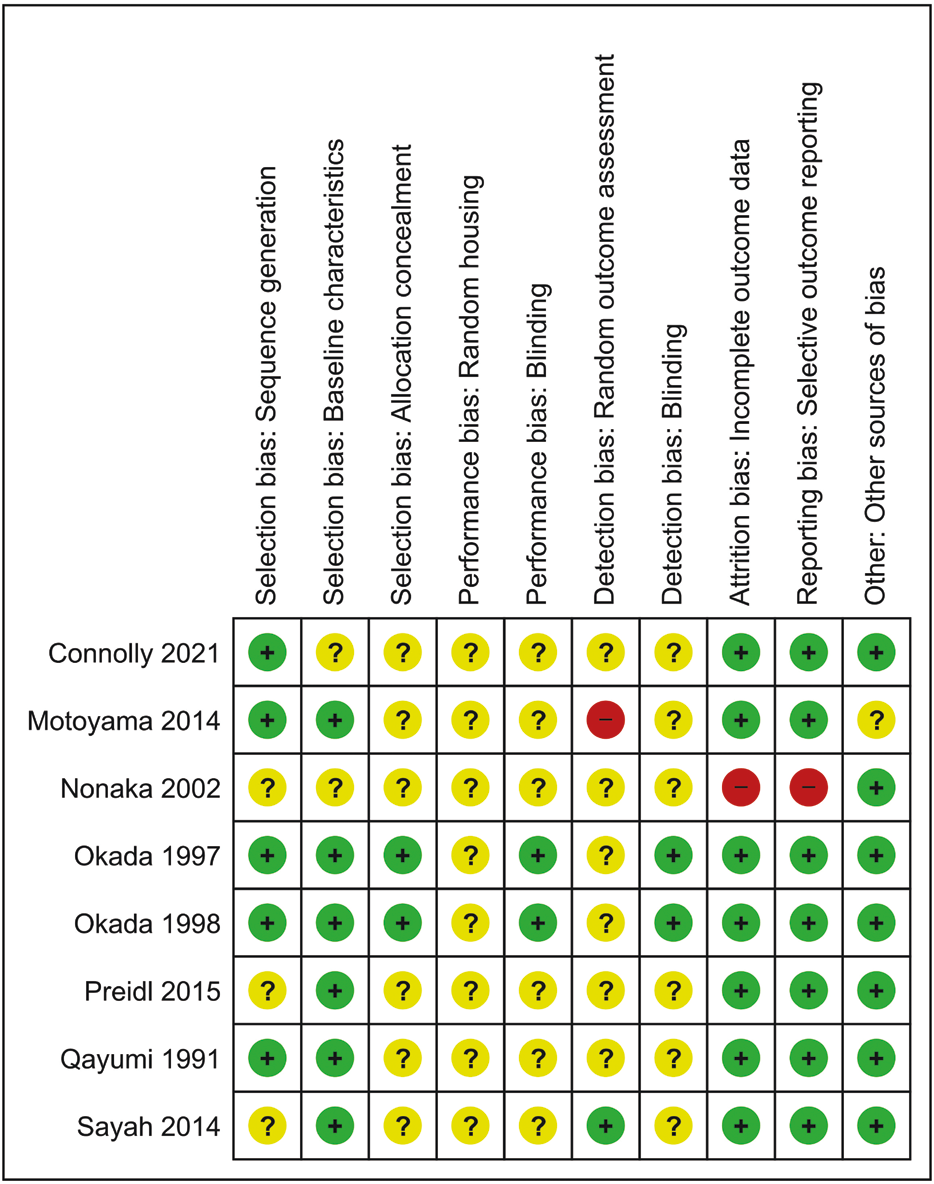
Risk of bias assessment based on SYRCLE’s risk of bias tool for animal studies. + refers to an article with a low risk of bias, ? refers to unclear risk of bias, and - refers to a high risk of bias.

In total, 9 studies were included in the review. Six small animal studies, 2 large animal studies, and 1 human study were included in this review. The included studies are summarized in Table 1 and outcomes of interest are reported in Table 2. Among the included studies, the majority of studies tended to have unclear or high risk related to blinding and randomization, which were infrequently explicitly reported. Selection bias, attrition bias, and reporting bias were generally low (Figure 1).

**TABLE 1.** Summary of included studies

Study name	Model/population	Study design	Intervention	Outcomes
Ex vivo lung perfusion				
Motoyama 2014^10^	Male Lewis rats undergoing EVLP and en bloc heart-lung transplantation	Rats were euthanized and underwent warm ischemia for 120 min and were then placed on ex vivo perfusion either with plasmin treatment for thrombosis or without	Beating heart control: 5 Untreated thrombosis: 7 Treated thrombosis (plasmin administration): 7	Measures of fibrinogen degradation products, PVR, lung weight gain, airway compliance, and histologic analyses
Nonaka 2002^29^	Guinea pig donor, Wistar rat heart-lung transplant recipients	Heart-lung blocks were excised from guinea pigs and perfused with either guinea pig blood, rat blood, or rat blood with an inhibitor of platelet aggregation (SH)	Rat organs perfused with rat blood: 6 Guinea pig organs perfused with rat blood: 6 Guinea pig organs perfused with rat blood and SH (platelet inhibition): 6	Levels of IgM and IgG were measured along with histological analyses of the lung samples
Connolly 2021^30^	Pigs with knockout of α1,3 galactosyltransferasegene (*GGTA1*; *GTKO*) and expressing human CD46 (hCD46), or GTKO.hCD46. Pig lungs perfused on EVLP	Pig lungs were perfused on EVLP with human blood, with the experimental group modified to exhibit humanized vWF	Control: 5 Pig lungs with humanized vWF: 5	Measures of vWF with Western blot and ELISA, Factor VIII activity, and immunohistochemistry
Lung transplantation				
Preidl 2015^31^	Mice undergoing tracheal transplantation	Trachea from mice were excised and transplanted into other mice. Mice that received the transplant were given low or high doses of clopidogrel	Control: 5 Low-dose clopidogrel: 5 High-dose clopidogrel: 5 Everolimus: 5 Tacrolimus: 5 Low-dose clopidogrel with everolimus: 5 Low-dose clopidogrel with tacrolimus: 5 High-dose clopidogrel with everolimus: 5	Analyses of interest included platelet aggregation, alloantibody detection, histology, immunohistochemistry, and analysis of intragraft mRNA expression
Sayah 2014^32^	Mouse model with treated lung ischemia	Mice had lung ischemia induced with hilar clamping. The ischemia was treated with aspirin and DNase-l. the treatment, the left lungs were excised and transplanted	Group 1: Hilar clamping Group 2: procured lungs with CSP	Neutrophil extracellular traps were imaged with immunofluorescence microscopy. Measures were also taken of plasma thromboxane and pO_2_ after transplantation
Okada 1997^33^	Rats undergoing lung transplantation	Ischemia was induced in rat lungs by preservation at 4 °C for 0, 6, or 24 h after which they were transplanted into other rats	Ischemia time 0 h: 5 6 h: 6 24 h: 6	Lung weight gain, right-to-left blood flow ratio, histopathology, and immunofluorescence staining
Okada 1998^34^	Rats undergoing lung transplantation	Ischemia was induced in rat lungs by preservation at 4 °C for 6 h or 24 h after which they were transplanted into other rats. Berprost sodium (antiplatelet medication) was administered to the experimental group	Control 6 h: 7 Beraprost sodium 6 h: 5 Control 24 h: 6 Beraprost sodium 24 h: 5	Lung weight gain, right-to-left blood flow ratio, histopathology, and immunofluorescence staining
Qayumi 1991^35^	Porcine heart-lung transplantation model	Porcine heart-lung transplantation model with ischemia induced by preservation times. Treatments included deferoxamine (iron chelating agent) and CV-3988 (platelet-activating factor antagonist)	Control: 7 Defuroxamine: 7 CV-3988: 7	Lung edema formation, pulmonary function, pH, CO_2_ tension, PVR, and pO_2_
Sternberg 2008^36^	Human patients who underwent lung transplantation or thoracotomy for nontransplant indications	Prospective clinical study comparing markers of platelet activation in patients who underwent lung transplantation or thoracotomy for nontransplant indications	Control: 10 Lung transplant: 7	Platelet-leukocyte conjugates measured by flow cytometry

CSP, cold static preservation; EVLP, ex vivo lung perfusion; PVR, pulmonary vascular resistance; SH, sarpogrelate hydrochloride; vWF, von Willebrand factor.

**TABLE 2.** Outcomes of included studies.

Study name	Physiologic parameters	Measures of platelet activity	Other
Ex vivo lung perfusion			
Motoyama 2014^10^	Plasmin administration was associated with lower PVR, reduced edema formation, improved compliance, and higher PaO_2_	Higher rates of fibrinogen degradation products in the plasmin group	Histologic analyses revealed more edema formation, hemorrhage, and apoptosis in the nontreated group compared with the plasmin group
Nonaka 2002^29^	–	–	Reduced IgM deposits with SH, suggesting a reduced hyperacute rejection response
Connolly 2021^30^	Lung perfusion parameters, including PVR, were similar between groups	Reduced platelet sequestration with humanized vWF when exposed to human blood	–
Lung transplantation			
Preidl 2015^31^	–	Mice treated with low or high doses of clopidogrel had lower rates of platelet aggregation compared with controls	Reduced rates of luminal obliteration, cellular infiltration, and circulating donor-specific antibodies with either immunosuppression or clopidogrel, and the least luminal obliteration with a combination of the 2
Sayah 2014^32^	Reduced rates of lung injury and improved oxygenation with aspirin administration	Levels of platelet-specific markers in the lung tissue and thromboxane in plasma and bronchoalveolar lavage fluid were reduced with the administration of aspirin	Aspirin administration reduced neutrophil extracellular trap formation and measures of lung injury, including bronchoalveolar lavage fluid neutrophilia and protein concentration
Okada 1997^33^	Blood flow ratio decreased in proportion to ischemia time. Lung weight gain also increased proportionally to the ischemia time	Platelet accumulation was proportional to the extent of pretransplant ischemia	Increased ischemia time was also associated with intra-alveolar edema, hemorrhage, and capillary congestion
Okada 1998^34^	Increased lung blood flow and reduced edema formation in rats that received beraprost sodium	Significant reductions in platelet accumulation in the rats that received beraprost sodium	Capillary congestion was more often identified in the control group compared with the experimental group
Qayumi 1991^35^	Reduced edema formation identified higher PaO_2_, reductions in acidosis, and reduced PVR with CV-3988	–	–
Sternberg 2008^36^	–	Markers of platelet activation (P-selectin and soluble CD40 ligand levels) and an increase in circulating platelet–monocyte conjugate fluorescence were increased posttransplant compared with nontransplant thoracotomy	–

PVR, pulmonary vascular resistance; SH, sarpogrelate hydrochloride; vWF, von Willebrand factor.

### Platelet Activation During EVLP

In the study by Motoyama et al,^10^ they randomly assigned male Lewis rats to 1 of 3 groups undergoing EVLP and heart-lung bloc transplantation: control with a beating heart, untreated thrombosis, and treated thrombosis with plasmin administration. In the control group, rats received heparin during organ procurement, whereas in the other 2 groups, they did not. After EVLP and cold static preservation for each group, the lungs were reperfused on an EVLP circuit using blood from donor rats. The authors found higher rates of fibrinogen degradation products in the plasmin group than in the non-plasmin group. This corresponded to a lower PVR, reduced edema formation, improved compliance, and a higher PaO_2_ in the plasmin group compared with the untreated thrombosis group.^10^

Nonaka et al^29^ investigated the impact of a platelet aggregation inhibitor on IgM deposition and hyperacute rejection in a guinea pig-to-rat lung transplant model. The organs were divided into 3 groups: rat organs perfused with rat blood, guinea pig organs perfused with rat blood, and guinea pig organs perfused with rat blood with sarpogrelate hydrochloride (an inhibitor of platelet aggregation; SH group). After exposures, the authors identified a reduction in IgM deposition in the SH group compared with the others, suggesting a reduced hyperacute rejection response.^29^

At the site of endothelial injury, the release of von Willebrand factor enhances the adhesion of platelets, and as a result, it is thought to play a role in arterial thrombus formation during EVLP and lung transplantation.^37,38^ A study by Connolly et al^30^ described the importance of platelet activation in an EVLP setting. The authors genetically modified pigs to exhibit humanized von Willebrand factor in an attempt to correct for platelet aggregation during EVLP using pig lungs and human blood. In the lungs expressing humanized von Willebrand factor, there was a reduction in platelet sequestration, preventing inappropriate platelet aggregation.^30^

### Platelet Activation During Lung Transplantation

Preidl investigated the influence of clopidogrel, an antiplatelet agent that works by irreversibly binding to platelets, on posttransplant obliterative bronchiolitis. The authors transplanted trachea from donor mice to recipient mice. The mice were divided into groups that were treated with high or low doses of clopidogrel with or without immunosuppression with everolimus or tacrolimus. This study found reduced rates of luminal obliteration for groups receiving either immunosuppression or clopidogrel, with the least luminal obliteration found in those treated with a combination of the 2. Cellular infiltration with CD4^+^ T cells, CD8^+^ T cells, and macrophages was reduced with clopidogrel, with a further reduction observed when combined with immunosuppression. Additionally, lower rates of intragraft proinflammatory cytokine production were identified in mice treated with clopidogrel. Finally, clopidogrel with or without immunosuppression resulted in reduced levels of circulating donor-specific alloantibodies.^31^ Although not necessarily focusing on lung transplantation, the use of clopidogrel after tracheal transplantation provides important insight into the role of platelet activation and thrombosis in combination with immune activation on obliterative bronchiolitis after transplantation.

Sayah et al^32^ hypothesized that a related pathway involving immune cells resulting in platelet activation contributes to PGD post–lung transplant. The authors described their study, which included 2 models using mice. In their first model, they induced lung ischemia through hilar clamping. In their second model, they procured lungs and preserved them with cold static preservation to induce ischemia. In their study, aspirin was used for platelet inhibition and l was used to disrupt neutrophil extracellular traps. The authors noted increased neutrophil extracellular traps, platelet activation, and intrapulmonary accumulation of platelets in the lung transplant group. In the mice who received aspirin, they identified reduced rates of lung injury and improved oxygenation.^32^

Okada et al^33^ conducted 2 studies investigating platelet accumulation after lung transplantation in rats. In their first study, heart-lung blocs were procured and stored at 4 °C for 0, 6, and 24 h to cause varying degrees of ischemic injury. The left lung was then divided from the bloc and transplanted with a reperfusion period of 1 h. They evaluated lung injury through the determination of preferential pulmonary blood flow as well as histological evaluation for edema, hemorrhage, and platelet aggregation. They found that platelet accumulation and measures of lung injury were proportional to the extent of pretransplant preservation and ischemia. Platelet accumulation was also proportional to blood flow ratio, levels of lung weight gain, and alveolar hemorrhage.^33^

In a follow-up study, Okada et al performed a similar experiment in which heart-lung blocs from donor rats were preserved for 6 or 24 h and the left lung was transplanted and reperfused for 1 h. One group received beraprost sodium, an agent with antiplatelet properties, whereas the other did not. The authors found significant reductions in platelet accumulation, increased lung blood flow, and reduced edema formation in the rats that received beraprost sodium at 6 h of ischemia. These findings were attenuated in the group that underwent 24-h preservation before transplantation.^35^

Qayumi et al performed heart-lung transplantations in a porcine model to create a model for ischemia/reperfusion injury. They took heart-lung blocs and subjected them to 4 h and 45 min of ischemia. They then transplanted the organs into recipient pigs and divided them into 3 groups: group A (control), group B, which received deferoxamine, and group C, which received the platelet-activating factor antagonist CV-3988. Deferoxamine is an iron chelating agent, which is proposed to inhibit the formation of oxygen-free radicals and mitigate ischemia/reperfusion injuries. The authors identified reductions in edema formation for group C compared with the other groups. They also found higher rates of PO_2_ in postreperfusion arterial blood gas, reductions in acidosis, and reduced PVR for group C.^35^

In a prospective clinical study, Sternberg et al^36^ reviewed markers of platelet activation in 7 patients who had undergone lung transplantation, with 10 patients who received a thoracotomy for another indication serving as controls. Markers of platelet activation, including P-selectin and soluble CD40 ligand levels, increased posttransplant compared with nontransplant thoracotomy. Furthermore, the authors identified an increase in circulating platelet–monocyte conjugate fluorescence post–lung transplantation.^36^

## DISCUSSION

Lung transplantation remains the gold standard for patients with end-stage lung disease. Additionally, EVLP is an exciting technology with the potential to improve patient outcomes by increasing donor lung availability and improving the quality of transplanted organs. However, both continue to be limited. EVLP is limited in maximum duration, with edema formation and graft dysfunction playing central roles in the limited longevity of lungs preserved on EVLP devices.^13,14,23-27,39^ Several factors have been suggested to contribute to edema formation and graft dysfunction, including an inflammatory cascade during EVLP as well as the development of arterial thrombosis, both of which likely play a role.^8,10,11,27^ Similarly, after lung transplantation, PGD occurs secondary to alveolar damage and ischemia/reperfusion injuries, resulting in edema formation, hypoxemia, and portending poor long-term outcomes.^11,27^ Although these conditions are certainly multifactorial, platelet aggregation and thrombosis have been infrequently investigated and addressed.

PGD after lung transplantation is multifactorial, including the influence of ischemia/reperfusion injury and inflammation.^8,9,11,27^ EVLP has previously been demonstrated to result in reduced rates of inflammation through reductions in cold ischemia times, buffered perfusate, and pharmacological mediators of inflammation and immune activation.^27^ Although lower than in traditional lung transplantation, inflammation still occurs as a result of endothelial injury, the period of ischemia, and blood contact with the perfusion circuit. An immunologic response and platelet activation occur secondary to inflammation during EVLP before transplantation.^27^ This mechanism is analogous to those described in lung transplantation and has been proposed to result in distal vessel thrombosis and subsequent graft dysfunction. The availability of platelets in the perfusate depends on whether a cellular or acellular perfusate is used in the EVLP system, although there is potential for thrombosis during procurement and after transplantation in cases where platelets are not present in the EVLP circuit. In a lung transplantation setting, several factors are proposed to result in early graft dysfunction. Ischemia during organ procurement due to both a lack of blood flow, platelet activation, and thrombosis results in cell damage and the release of cytotoxic enzymes.^8,9,11,27^ During the reperfusion phase, reactive oxygen intermediates are generated and platelet and immune cell activation occurs. Platelet adhesion and aggregation at the site of endothelial injury are facilitated by von Willebrand factor, which is upregulated by several of the aforementioned conditions seen during lung transplantation, including hypoxia, proinflammatory cytokine production, and the presence of reactive oxygen intermediates, among others.^8,11,27^ This, in turn, leads to the formation of microthrombus, microvascular vasoconstriction, capillary congestion, endothelial damage, cytokine and complement activation, increased vascular permeability, and edema formation.^8,11,27^ This inflammatory state leads to additional thrombosis and fibrin deposition, with platelets adhering to the endothelium, causing further tissue injury and the release of free radicals, vasoactive mediators, and inflammatory mediators, in turn eliciting further inflammation.^8,11,27^ Furthermore, the inflammatory immune response of the recipient activates platelets along with the remainder of the inflammatory cascade, resulting in lung injury.^9,11^

Several studies published in preceding decades have attempted to identify the role of platelet activation and aggregation in the development of graft dysfunction both in EVLP and lung transplantation.^11^ Small animal and large animal trials have found that platelet aggregation and distal arterial thrombosis lead to pulmonary congestion, inflammation, and edema formation.^10,30–33,35,40^ Only a single human study was identified in this review, which found markers of increased platelet activation after lung transplantation.^32^ The data available in the literature on this topic reinforce the notion that platelet activation may play a role in some of the current limitations of EVLP and lung transplantation, although these data are largely limited to animal models and the paucity of data from human participants limits our ability to understand the impact of platelet aggregation and arterial thrombosis in clinical settings of EVLP and lung transplantation. This review consolidated the information available on this topic, while also identifying areas that require further investigation. Although limited, the available literature has demonstrated an association between platelet activation and various antiplatelet agents in reducing lung injury and improving function in various animal models. The similar findings in a variety of settings suggest there is an association between platelet activation and lung function in the settings of EVLP and lung transplantation, and these findings should encourage additional investigation into this field, given the potential impact on clinical outcomes if this association is conclusively proven. Future studies should aim to use clinically available antiplatelet agents, additional animal models, and human participants through retrospective analyses and prospective trials to confirm the translation of these findings into clinical settings. In clinical settings, the administration of antiplatelet agents after lung transplantation may help to prevent arterial thrombosis, although this must certainly be balanced with the risk of bleeding after an invasive operation. The optimal selection of antiplatelet agents and their dosing is yet to be determined.

### The Role of Antiplatelet Agents in EVLP and Lung Transplantation

There are currently no guideline recommendations regarding antiplatelet use in lung transplantation, likely owing to the limited evidence regarding this topic. As highlighted by this review, few studies have investigated the impact of platelet activation in EVLP and lung transplantation settings, although those that have investigated this topic have found evidence suggesting that platelets may play a role in lung dysfunction during EVLP and transplant, as well as the potential benefit of antiplatelet therapy in these settings. However, the available evidence is insufficient to conclusively determine the optimal use of antiplatelet agents in EVLP and transplant. Several key questions remain that must be addressed.

First, in the setting of EVLP, the role of antiplatelet agents will likely vary depending on the protocol used. In EVLP using whole blood, investigations into the impact of antiplatelet agents are feasible given the circulating platelet volume. In acellular EVLP or EVLP circuits using washed blood without platelets, the role of antiplatelets is less clear. In these settings, there is likely an insignificant volume of circulating platelets to justify the use of antiplatelet, although this is not certain. Platelet activation during procurement and ischemia may result in platelet aggregation and distal arterial thrombosis before the establishment of EVLP. Furthermore, it is likely that endothelial injury sustained during procurement and cold ischemic time, inflammation from cardiopulmonary bypass, extracorporeal membrane oxygenation, and surgical stimulation, along with reperfusion injury, may further stimulate platelet activation post-EVLP and transplant. Therefore, antiplatelet agents may still play a role in the setting of acellular EVLP. Pretreatment of the donor with antiplatelet therapy, along with postoperative antiplatelet use, may help to prevent platelet aggregation. Certainly, there is concern with antiplatelet use and postoperative bleeding that requires consideration. Only with robust evidence supporting, or not supporting, the use of antiplatelet agents in the EVLP and lung transplantation settings can the risks and benefits of this intervention be known.

### Limitations

There are limitations to this review. These include heterogeneous studies with limited numbers that hinder our ability to aggregate data for analysis. Furthermore, the vast majority of studies were performed in animal models with only 1 prospective study of human participants.

## CONCLUSIONS

Platelet activation in the setting of EVLP and lung transplantation has been demonstrated in numerous settings. The activation of platelets results in distal vessel thrombosis and has been associated with graft dysfunction. In a limited number of investigations, the use of antiplatelet agents was associated with reduced rates of platelet activation and accumulation, lower rates of lung injury and edema formation, as well as improved lung function on EVLP or during lung transplantation. Although the available evidence supports an association between platelets and lung function in the setting of EVLP and lung transplantation, the available literature is limited and heterogeneous. Future investigations into this topic are required to conclusively determine the role of antiplatelet agents in EVLP and lung transplantation.
